# Editorial: Tumor Microenvironment and Resistance to Current Therapies

**DOI:** 10.3389/fonc.2019.01131

**Published:** 2019-11-14

**Authors:** Ahmed Lasfar, Murugabaskar Balan, Karine A. Cohen-Solal, Andrew Zloza

**Affiliations:** ^1^Rutgers Cancer Institute of New Jersey, Rutgers, The State University of New Jersey, New Brunswick, NJ, United States; ^2^Department of Pharmacology and Toxicology, Ernest Mario School of Pharmacy, Rutgers, The State University of New Jersey, New Brunswick, NJ, United States; ^3^Harvard Medical School, Boston Children's Hospital, Boston, MA, United States; ^4^Rush University Medical Center, Chicago, IL, United States

**Keywords:** cancer resistance, tumor—targeting, tumor microenvironment, immunotherapy, therapy resistance, cancer cell signaling, targeted therapy

The tumor microenvironment (TME) constitutes an important component of any cancer. The histology of TME consists of a series of normal resident cells and a variety of recruited cells, which are involved in complex and dynamic interactions with cancer cells (Figure 1). These interactions, which occur via released factors or cell-to-cell contact, are fundamental in tumor-induced suppression and metastatic dissemination of cancer cells, ultimately leading to morbidity and/or mortality for the majority of cancer patients. Considerable progress has been made in understanding the mechanisms by which the TME contributes to the inhibition or promotion of cancer, enabling the emergence of a range of novel targeted therapies. In addition to stroma-targeted strategies, checkpoint inhibitor-based immunotherapy has emerged as a new treatment of choice for many advanced cancers. However, many cancer patients remain resistant to current therapies, necessitating the development of more innovative therapeutic strategies based on the identification of new targets and combining drugs that could counteract resistance. In the present issue, “Tumor microenvironment and resistance to current therapies,” authors highlight this aspect in the included articles describing innovative and impactful studies.

**Figure 1:**
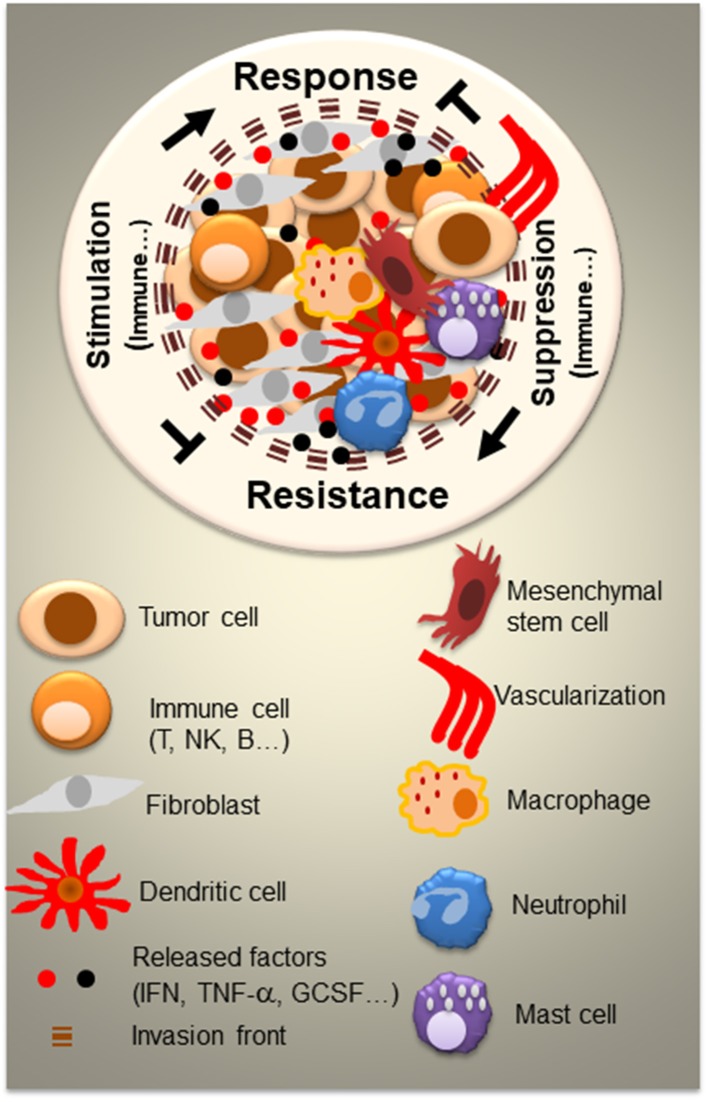
Tumor microenvironment (TME) and the complexity of interactions between resident and recruited normal cells and tumor cells. Besides tumor cells, the tumor TME consists of a variety of normal cells and factors which can be serves as mediators for both cancer control and cancer outgrowth. Normal cells are either resident cells or recruited cells. They vary from each cancer type and level of cancer progression. In large part, they consist of immune cells (T, B, and NK cells, mast, dendritic cells, macrophages, and neutrophils) and non-immune cells such as fibroblasts and mesenchymal stem cells. All of the cells release and respond to a variety factors (IFN, TNF-α, GCSF,…) and engage in cell-to-cell contact. Cell signaling within the TME represents a crucial key for the design of successful therapeutic strategies.

In this Research Topic, diverse facets of the TME are addressed, including different tumor types and tissue specificity of TME (Figure 1). Recently, the TME has emerged as a key player in both drug efficacy and chemoresistance, impelling the development of novel therapeutic strategies. Despite constant progress in antitumor drug development, multidrug resistance (MDR) is still a major challenge for effective patient treatment. Sachs et al., describe a novel method for overcoming this problem by demonstrating, in different cancer models, the ability of new small molecules to reverse chemotherapy resistance. Munoz et al., address the problem of chemoresistance of glioblastoma multiforme (GBM), a fatal malignancy of the central nervous system. They show that MiR-93 and−193 are specifically expressed in temozolomide (TMZ) resistant glioblastoma cells (GBM), including resistant neurospheres from a patient with TMZ resistance. Part of the resistance occurs by miR-93 and−193 decreasing Cyclin D1 to reduce GBM cycling. This may open up avenues for new therapeutic approaches to reverse such chemoresistance (Munoz et al.). Besides chemoresistance in GBM, expansion of residual glioma cells is also a challenge in the context of chemotherapy. Work by Tsidulko et al., indicates that conventional anti-glioblastoma therapy such as TMZ affects proteoglycan structure and composition of normal brain tissue. Proteoglycan alteration may be involved in brain extracellular matrix (ECM) deterioration and the development of residual glioma cells (Tsidulko et al.).

Although immune checkpoint blockade therapy has afforded new hope for the durable treatment of many cancers, it has not provided yet substantial benefit to patients with glioblastoma. Utilizing a series of epidemiological, genome expression, and immunome databases, Ladomersky et al., demonstrate a significant association between advanced age of patients and immunosuppression in the circulation and central nervous system. Based on their findings, the authors propose that normal human aging suppresses immune surveillance and immunotherapeutic efficacy against glioma, and thus, contributes to GBM initiation and progression (Ladomersky et al.).

Using a lung cancer model, Yang et al., focus their study on bronchoalveolar lavage fluid-derived exosomes, released in the TME and demonstrate their role in fueling inflammation and tumor invasiveness via mast cells/neutrophils activation and cytokines release in TME. In a gastric cancer study, Zhao et al., analyze the role of NEDD9 in cancer cell migration under hypoxia. They demonstrate that NEDD9 regulates MICAL1, which facilitates hypoxia-induced gastric cancer cell migration in a Rac1-dependent manner (Zhao et al.).

Tuo et al., address the role of the cytoplasmic isoform of phosphoenolpyruvate carboxykinase (PCK1) in the progression of hepatocellular carcinoma (HCC). PCK1 is a rate-limiting enzyme in gluconeogenesis which occurs mainly in the liver. The authors find that the expression of PCK1 is down regulated in HCC and associated with poor outcome. In hepatoma cells, the authors demonstrate that reactive oxygen species (ROS) production and nuclear translocation of Nrf2 and thioredoxin reductase 1 (TXNRD1) are suppressed during PCK1 overexpression. Furthermore, the authors show that targeting the TXNRD1 antioxidant pathway sensitizes PCK1-knockout hepatoma cells to sorafenib treatment *in vitro* (Tuo et al.).

The role of natural products in the modulation of the TME is likewise addressed in this Research Topic. Hu et al., investigate the effect of berberine (BBR) in gastric cancer (GC). BBR is a natural isoquinoline alkaloid, presumably involved in lipid metabolism and glucose homeostasis by regulating the expression of HNF4α. The authors show that BBR inhibits the proliferation, invasion, and migration of GC cell lines, and thus reduces GC tumor growth *in vivo*. The antitumor effect of BBR shown appears to involve the AMPK-HNF4α-WNT5A signaling pathway (Hu et al.). Using scutellarin, an active flavone extracted from *Erigeron breviscapus* Hand-Mazz (EBHM), Sun et al., investigate cisplatin resistance in non-small cell lung cancer (NSCLC). The authors find that scutellarin sensitizes tumor cells to cisplatin by enhancing apoptosis and autophagy via downregulation of p-AKT and c-Met in autophagy and caspase-3-dependent apoptosis. They suggest that the combination of cisplatin and scutellarin may be a novel therapeutic strategy for patients with NSCLC (Sun et al.).

The impact of DNA damage on TME is also investigated. Tirado-Hurtado et al., highlight the role of DNA Damage Inducible Transcript 4 (DDIT4) in cancer. The DDIT4 gene is expressed under stress situations, turning off the metabolic activity triggered by the mammalian target of rapamycin (mTOR). The authors propose targeting DDIT4 for the development of novel drugs that could be more specific and efficient than current mTOR inhibitors (Tirado-Hurtado et al.).

Mesenchymal stem cells (MCSs) play important role in the TME (Figure 1). Chulpanova et al., address the use of MCSs for drug delivery in oncology. The authors focus on MSC and tumor interactions, which are crucial for cancer control. They also describe novel therapeutic strategies using MSCs and MSC-derived membrane microvesicles for cancer therapy (Chulpanova et al.).

In conclusion, investigation of TME continues to be a vital focus toward the elaboration of novel strategies that produce more effective treatments for localized cancers and metastases. Findings presented in this Research Topic have the potential to make a major impact on this field and to inspire further discoveries.

## Author Contributions

AL drafted the manuscript. AL, MB, KC-S, and AZ critically reviewed the manuscript for important intellectual content and approved it for publication.

### Conflict of Interest

AL and KC-S are Founders and Shareholders of Lambda Pharmaceuticals. The remaining authors declare that the research was conducted in the absence of any commercial or financial relationships that could be construed as a potential conflict of interest.

